# Supplementary material for: Women’s empowerment and contraceptive use: Recent evidence from ASEAN countries
Source: PLoS One. 2023 Jun 27;18(6):e0287442. doi: 10.1371/journal.pone.0287442 (PMC10298759; doi:10.1371/journal.pone.0287442)
Supplement: Attachment — Submitted filename: Comments on PONE-D-22-02451.docx [file pone.0287442.s001.docx]

Comments on PONE-D-22-02451

Studies of the relationship of women’s empowerment and contraceptive use are important, and needed generally and in countries of the ASEAN region. The authors of this manuscript leverage publicly available Demographic and Health Survey data for several ASEAN countries to examine the relationship of four variables they use to operationalize women’s empowerment (labor force participation; disagreement with reasons for wife beating; decision-making power over household issues; and ‘knowledge level’) with women’s current contraceptive use. I hope my comments below contribute to strengthening the contribution of this manuscript to the field.

Background

1. The background section would benefit from a clear definition of women’s empowerment, with reference to the appropriate theoretical literature. I recommend Kabeer (1999) as a seminal reference to cite.
2. This reference will help the authors distinguish conceptually between the ‘resources’ measures of empowerment they include (e.g., labor force participation) and the ‘agency’ measures they include (e.g., attitudes about wife beating; influence in decisions, and knowledge). Thus, the variables they use to operationalize ‘women’s empowerment’ should be conceptualized according to the ‘resources’ and ‘agency’ components of women’s empowerment.
3. Relevant references are missing from the background and should be added. A key reference is a systematic review of the literature on women’s agency and contraceptive use, by Laurie James-Hawkins and colleagues: James-Hawkins, L., Peters, C., VanderEnde, K., Bardin, L., & Yount, K. M. (2018). Women’s agency and its relationship to current contraceptive use in lower-and middle-income countries: A systematic review of the literature. Global Public Health, 13(7), 843-858. More nuanced discussion of the empirical literature on women’s empowerment and contraceptive use also may be useful.

Methods

1. More information about each sample is needed, for example response rates and any missing data on variables of interest and how those missing data are handled in the analysis.
2. The variable ‘knowledge level’ needs to be defined more clearly in the methods and throughout the manuscript (knowledge level of what?).
3. The variable for ‘contraceptive use’ also needs to be defined more clearly in the methods and throughout the manuscript (e.g., what methods were asked about in each survey; were the methods asked about the same across surveys? What does ‘current’ mean, e.g., at the time of the survey?)
4. Why is age included separately in the ‘conceptual framework’ (which anyway should go into the background, not the methods section)? Is age rather a covariate?
5. Why is women’s schooling not included as a resource for their empowerment?
6. The variables for women’s ‘agency’ (attitudes about wife beating, decision-making influence, knowledge) need to be described in much more detail. How many items are available each construct? Are the number of items the same across countries (they should be). What are the response options for each construct? How response options were coded/recoded for the analysis? How specifically were these variables constructed for the analysis? What does ‘low, medium, high’ mean for each variable, and is this an appropriate way to model these measures (what is the theoretical rationale for doing so?)
7. The Analysis section should be separate from the Variables section in the Methods, and much more detail should be provided on the a) descriptive analyses performed in the pooled and country-specific samples, b) process for constructing each of the agency scales/variables and assessing their item and scale distributions and alpha reliability within and across countries, and c) sequential regression analysis (unadjusted and adjusted models in the pooled and/or stratified samples). Also if women’s LFP is a resource for empowerment, is a hierarchical model (unadjusted models, resources for empowerment, resources and agency for empowerment to assess mediation) more appropriate on theoretical grounds? Also, how was the multi-stage cluster sample design handled for each country?

Results and Discussion

1. Without the above detail, it is difficult to interpret the accuracy or reasonableness of the findings.
2. After the above methods issues are sorted, I recommend that the Discussion section also include a thoughtful discussion of the measures of empowerment (resources and/or agency) that were not included but that may be relevant for all countries or for specific countries. For example, is women’s freedom of movement or lack thereof relevant for some ASEAN countries if not for others? What are the implications for the results of leaving out important cross-country or country-specific measures of women’s resources and/or agency? If you could, what alternative measures might you use for constructs of agency that were not associated with contraceptive use in the final models? Finally, what are the potential pitfalls of recommending that women’s labor force participation is important for their empowerment without certain qualifications (e.g., implications for their time-use agency; triple burden of paid labor, unpaid domestic labor, and unpaid care work)

Thank you for the opportunity to review this manuscript. I hope the above comments are helpful.
