# Supplementary material for: Women’s empowerment and contraceptive use: Recent evidence from ASEAN countries
Source: PLoS One. 2023 Jun 27;18(6):e0287442. doi: 10.1371/journal.pone.0287442 (PMC10298759; doi:10.1371/journal.pone.0287442)
Supplement: Attachment — Submitted filename: response to reviewer final.docx [file pone.0287442.s002.docx]

**AUTHORS’ RESPONSES TO REVIEWERS**

**Academic Editor**

| **No** | **Reviewer’s Comments** | **Authors’ Responses** |
| --- | --- | --- |
| 1 | Please include the following items when submitting your revised manuscript:   - A rebuttal letter that responds to each point raised by the academic editor and reviewer(s). You should upload this letter as a separate file labeled 'Response to Reviewers'. - A marked-up copy of your manuscript that highlights changes made to the original version. You should upload this as a separate file labeled 'Revised Manuscript with Track Changes'. - An unmarked version of your revised paper without tracked changes. You should upload this as a separate file labeled 'Manuscript'. | Many thanks, we have prepared the three files as requested |
| 2 | 1. Please ensure that your manuscript meets PLOS ONE's style requirements, including those for file naming. The PLOS ONE style templates can be found at  <https://journals.plos.org/plosone/s/file?id=wjVg/PLOSOne_formatting_sample_main_body.pdf> and  <https://journals.plos.org/plosone/s/file?id=ba62/PLOSOne_formatting_sample_title_authors_affiliations.pdf> | Many thanks, we have ensured that the manuscript meets PLOS ONE's style requirements |
| 3 | 2. Thank you for stating in your Funding Statement:  Please provide an amended statement that declares *all* the funding or sources of support (whether external or internal to your organization) received during this study, as detailed online in our guide for authors at <http://journals.plos.org/plosone/s/submit-now>.  Please also include the statement “There was no additional external funding received for this study.” in your updated Funding Statement.  Please include your amended Funding Statement within your cover letter. We will change the online submission form on your behalf. | ” F.E. received funding support from the Ministry of Research, Technology and Higher Education of Indonesia during his post-doctoral degree. The funding source was not involved in study design, data collection, analysis or interpretation; in the writing of this report; or in the decision to submit the article for publication.” |
|  | Reviewer 1 |  |
| 5 | 1. Is the manuscript technically sound, and do the data support the conclusions?  The manuscript must describe a technically sound piece of scientific research with data that supports the conclusions. Experiments must have been conducted rigorously, with appropriate controls, replication, and sample sizes. The conclusions must be drawn appropriately based on the data presented.  Reviewer #1: Partly  Reviewer #2: Yes | Thank you for the comment |
| 6 | 2. Has the statistical analysis been performed appropriately and rigorously?  Reviewer #1: No  Reviewer #2: Yes | Thank you for the comment |
| 7 | 3. Have the authors made all data underlying the findings in their manuscript fully available?  The [PLOS Data policy](http://www.plosone.org/static/policies.action#sharing) requires authors to make all data underlying the findings described in their manuscript fully available without restriction, with rare exception (please refer to the Data Availability Statement in the manuscript PDF file). The data should be provided as part of the manuscript or its supporting information, or deposited to a public repository. For example, in addition to summary statistics, the data points behind means, medians and variance measures should be available. If there are restrictions on publicly sharing data—e.g. participant privacy or use of data from a third party—those must be specified.  Reviewer #1: Yes  Reviewer #2: Yes | Thank you for the comment |
| 8 | 4. Is the manuscript presented in an intelligible fashion and written in standard English?  PLOS ONE does not copyedit accepted manuscripts, so the language in submitted articles must be clear, correct, and unambiguous. Any typographical or grammatical errors should be corrected at revision, so please note any specific errors here.  Reviewer #1: Yes  Reviewer #2: Yes | Thank you for the comment |
| 9 | 5. Review Comments to the Author  Please use the space provided to explain your answers to the questions above. You may also include additional comments for the author, including concerns about dual publication, research ethics, or publication ethics. (Please upload your review as an attachment if it exceeds 20,000 characters)  Reviewer #1: Comments on PONE-D-22-02451  Studies of the relationship of women’s empowerment and contraceptive use are important, and needed generally and in countries of the ASEAN region. The authors of this manuscript leverage publicly available Demographic and Health Survey data for several ASEAN countries to examine the relationship of four variables they use to operationalize women’s empowerment (labor force participation; disagreement with reasons for wife beating; decision-making power over household issues; and ‘knowledge level’) with women’s current contraceptive use. I hope my comments below contribute to strengthening the contribution of this manuscript to the field. | Thank you for the comment |
| 10 | **Background**  1. The background section would benefit from a clear definition of women’s empowerment, with reference to the appropriate theoretical literature. I recommend Kabeer (1999) as a seminal reference to cite. | Thank you very much. We add the reference in the background.  “Women's empowerment according to Kabir (1999) is the ability of women to make life choices which combines 3 interrelated dimensions including: resources (access, material and human and social resources); agency (decision-making processes, less measurable manifestations of agency such as negotiation, deception, and manipulation); and achievement (welfare outcomes) (Kabeer, 1999a; Kabeer, 1999b).”   1. Kabeer, N. (1999). Resources, agency, achievements: Reflections on the measurement of women's empowerment. *Development and change*, *30*(3), 435-464. 2. Kabeer, N. (1999). *The conditions and consequences of choice: reflections on the measurement of women's empowerment* (Vol. 108, pp. 1-58). Geneva: UNRISD. |
| 11 | 2. This reference will help the authors distinguish conceptually between the ‘resources’ measures of empowerment they include (e.g., labor force participation) and the ‘agency’ measures they include (e.g., attitudes about wife beating; influence in decisions, and knowledge). Thus, the variables they use to operationalize ‘women’s empowerment’ should be conceptualized according to the ‘resources’ and ‘agency’ components of women’s empowerment. | Thank you very much. We add the reference in the background.  “The role of women's empowerment is measured by variables, namely labor force participation, disagreement to justification toward wife beating, decision-making power over household issues, and knowledge level (Phan, 2015;Sebayang, Efendi, and Astutik, 2017). Labor force participation is part of component of the resources. Disagreement to justification toward wife beating, decision-making power over household issues, and knowledge level variables are part of the agency component of women's empowerment roles (Kabeer, 1999a; Kabeer, 1999b).”   1. Phan L. Measuring women’s empowerment at household level using DHS data of four Southeast Asian countries. Soc Indic Res. 2016;126(1):359–78. 2. Sebayang SK, Efendi F, Astutik E. Women’s empowerment and the use of antenatal care services: analysis of demographic health surveys in five Southeast Asian countries. Women Health [Internet]. 2019 Apr;1–17. Available from: <https://www.tandfonline.com/doi/full/10.1080/03630242.2019.1593282> 3. Kabeer, N. (1999). Resources, agency, achievements: Reflections on the measurement of women's empowerment. *Development and change*, *30*(3), 435-464. 4. Kabeer, N. (1999). *The conditions and consequences of choice: reflections on the measurement of women's empowerment* (Vol. 108, pp. 1-58). Geneva: UNRISD. |
| 12 | 3. Relevant references are missing from the background and should be added. A key reference is a systematic review of the literature on women’s agency and contraceptive use, by Laurie James-Hawkins and colleagues: James-Hawkins, L., Peters, C., VanderEnde, K., Bardin, L., & Yount, K. M. (2018). Women’s agency and its relationship to current contraceptive use in lower-and middle-income countries: A systematic review of the literature. Global Public Health, 13(7), 843-858. More nuanced discussion of the empirical literature on women’s empowerment and contraceptive use also may be useful. | Thank you very much. We have added the reference in the background  “Research shows that there is a positive relationship between women's empowerment and reproductive health. Women's empowerment is associated with the use of female contraceptives in low- and middle-income countries. The results of the study also mention that understanding the causal impact of women's agency on contraceptive use, and will help inform policies and programs to increase contraceptive use (James-Hakins et al, 2018).”  James-Hawkins, L., Peters, C., VanderEnde, K., Bardin, L., & Yount, K. M. (2018). Women’s agency and its relationship to current contraceptive use in lower-and middle-income countries: A systematic review of the literature. *Global Public Health*, *13*(7), 843-858. |
|  | **Methods**  1. More information about each sample is needed, for example response rates and any missing data on variables of interest and how those missing data are handled in the analysis. | Thank you very much, we add the information in the methods.  “We used complete cases for all analysis. We used complete cases for all analysis. The total number of respondents were 10,403 for Cambodia, 31,495 for Indonesia, 6,047 for Myanmar, 12,815 for The Philippines, and 5,011 for Timor-Leste. DHS data represent a response rate of 98% of women for Indonesia, Cambodia, and The Philippines, 97% of women for Timor Lester and 96% of women for Myanmar.”   1. National Institute of Statistics. Cambodia Demographic and Health Survey 2014 [Internet]. 2015 [cited 2022 Oct 8]. Available from: <https://dhsprogram.com/pubs/pdf/fr312/fr312.pdf> 2. National Population and Family Planning Board. Indonesia Demographic and Health Survey 2017 [Internet]. 2018 [cited 2022 Oct 8]. Available from: <https://dhsprogram.com/pubs/pdf/FR342/FR342.pdf> 3. Statistics P, City Q. Philippines National Demographic and Health Survey 2017 Key Indicators Report. 2018; 4. General Directorate of Statistics Ministry of Planning and Finance and Ministry of Health. Timor-Leste Demographic and Health Survey 2016 [Internet]. 2017 [cited 2022 Oct 8]. Available from: <https://www.dhsprogram.com/pubs/pdf/FR329/FR329.pdf> 5. Ministry of Health and Sports, Nay Pyi Taw M. MYANMAR DEMOGRAPHIC AND HEALTH SURVEY 2015-2016 [Internet]. 2017 [cited 2022 Oct 8]. Available from: https://dhsprogram.com/pubs/pdf/FR324/FR324.pdf |
|  | 2. The variable ‘knowledge level’ needs to be defined more clearly in the methods and throughout the manuscript (knowledge level of what?). | Thank you very much, we added the information in the methods.  “The last component on women’s knowledge includes access to media (newspaper, radio, and television) and the highest level of formal educational. Access to media was divided to 3 categories, namely not at all, some of media, all of media. the highest level of formal educational was categorized into no education, primary, secondary and higher education.” |
|  | 3. The variable for ‘contraceptive use’ also needs to be defined more clearly in the methods and throughout the manuscript (e.g., what methods were asked about in each survey; were the methods asked about the same across surveys? What does ‘current’ mean, e.g., at the time of the survey?) | Thank you very much. We added the information in the methods  “Currently use of contraception was measured of percentage of women who currently use specific methods. Currently use the specific method (v312 = x), after being asked whether they or their partner are currently doing something or using any method to delay or avoid getting pregnant. Women who say they are pregnant are coded as not currently using any method.”   1. DHS. The DHS Program - DHS Survey Indicators - Family Planning [Internet]. [cited 2022 Oct 8]. Available from: <https://dhsprogram.com/data/dhs-survey-indicators-family-planning.cfm> 2. DHS. Current Use of Contraceptive Methods [Internet]. Available from: https://dhsprogram.com/data/Guide-to-DHS-Statistics/Current_Use_of_Contraceptive_Methods.htm |
|  | 4. Why is age included separately in the ‘conceptual framework’ (which anyway should go into the background, not the methods section)? Is age rather a covariate? | Thank you very much. Age is one of our covariate variables |
|  | 5. Why is women’s schooling not included as a resource for their empowerment? | Thank you very much. Women’s schooling was included in the component of women knowledge level. We added the information in the methods.  The last component on women’s knowledge includes access to media (newspaper, radio, and television) and the highest level of formal educational. Access to media was divided to 3 categories, namely not at all, some of media, all of media. the highest level of formal educational was categorized into no education, primary, secondary and higher education. |
|  | 6. The variables for women’s ‘agency’ (attitudes about wife beating, decision-making influence, knowledge) need to be described in much more detail. How many items are available each construct? Are the number of items the same across countries (they should be). What are the response options for each construct? How response options were coded/recoded for the analysis? How specifically were these variables constructed for the analysis? What does ‘low, medium, high’ mean for each variable, and is this an appropriate way to model these measures (what is the theoretical rationale for doing so?) | Thank you very much. We added the information in the methods. The number of items were the same across countries.  “We used Principal Component Analysis (PCA) to create empowerment components. PCA is adopted for creating women empowerment proxies in order to investigate the independent effects of women empowerment on certain health outcome (Sebayang, et al, 2020). These four components of women’s empowerment were calculated from 17 indicators from the DHS surveys using a principal component analysis. Components of labor force participation include six aspects: work in the last 12 months, for whom women work, women’s occupation, types of payment, working all years and earning more than husband. The second component of attitudes toward wife beating includes five aspects: neglecting children, going places without husband’s permission, arguing, refusing sex and burning food. The third component on decision making includes own health care, household purchasing, visiting family and husband’s earnings. “ |
|  | 7. The Analysis section should be separate from the Variables section in the Methods, and much more detail should be provided on the a) descriptive analyses performed in the pooled and country-specific samples, b) process for constructing each of the agency scales/variables and assessing their item and scale distributions and alpha reliability within and across countries, and c) sequential regression analysis (unadjusted and adjusted models in the pooled and/or stratified samples). Also if women’s LFP is a resource for empowerment, is a hierarchical model (unadjusted models, resources for empowerment, resources and agency for empowerment to assess mediation) more appropriate on theoretical grounds? Also, how was the multi-stage cluster sample design handled for each country? | Thank you very much. We revised it and we added information in methods  “The data analysis took into account the complex survey sampling design, which produced the weighted results. Respondents’ characteristics, the component of women’s empowerment, and Current use of Contraceptives as well as covariate variables were descriptively presented. The bivariate analysis with simple logistic regressions was carried out to perform the Crude Odds Ratio. Multiple logistic regression was used to examine the relationship between women’s empowerment and outcome variables adjusting for covariates variables regression with svy command to adjust for the DHS sampling design. Data were analyzed by STATA 14. “ |
|  | 2. After the above methods issues are sorted, I recommend that the Discussion section also include a thoughtful discussion of the measures of empowerment (resources and/or agency) that were not included but that may be relevant for all countries or for specific countries. For example, is women’s freedom of movement or lack thereof relevant for some ASEAN countries if not for others? What are the implications for the results of leaving out important cross-country or country-specific measures of women’s resources and/or agency? If you could, what alternative measures might you use for constructs of agency that were not associated with contraceptive use in the final models? Finally, what are the potential pitfalls of recommending that women’s labor force participation is important for their empowerment without certain qualifications (e.g., implications for their time-use agency; triple burden of paid labor, unpaid domestic labor, and unpaid care work)  Thank you for the opportunity to review this manuscript. I hope the above comments are helpful. | Thank you very much for this positive feedback, we have add more elaboration on the discussion section. |
|  | Reviewer #2: In figure(1), why Age in a separate box as age is a covariate and could not see the analysis with age. | Thank you very much. Age is covariate variables. We revise the figure  “These covariates included age (categorized into five-year groups, namely 15-19, 20-24, 25-29, 30-34, 35-39, 40-44, 45-49 years old),…” |
|  | Outcome is Current use of contraception and no need to present "not use" | Thank you very much, we added some information in the methods  “Currently use of contraception was measured of percentage of women who currently use specific methods. Currently use the specific method (v312 = x), after being asked whether they or their partner are currently doing something or using any method to delay or avoid getting pregnant. Women who say they are pregnant are coded as not currently using any method.” |
|  | The working definition for tertiles should be mentioned. | Thank you very much, we added some information in the methods  “We used Principal Component Analysis (PCA) to create empowerment components. PCA is adopted for creating women empowerment proxies in order to investigate the independent effects of women empowerment on certain health outcome (Astutik, Erni, et al, 2020). These four components of women’s empowerment were calculated from 17 indicators from the DHS surveys using a principal component analysis. Components of labor force participation include six aspects: work in the last 12 months, for whom women work, women’s occupation, types of payment, working all years and earning more than husband. The second component of attitudes toward wife beating includes five aspects: neglecting children, going places without husband’s permission, arguing, refusing sex and burning food. The third component on decision making includes own health care, household purchasing, visiting family and husband’s earnings. “ |
|  | One sentence for Analysis is not enough. | Thank you very much, we added some information in the methods  “The data analysis took into account the complex survey sampling design, which produced the weighted results. Respondents’ characteristics, the component of women’s empowerment, and Current use of Contraceptives as well as covariate variables were descriptively presented. The bivariate analysis with simple logistic regressions was carried out to perform the Crude Odds Ratio. Multiple logistic regression was used to examine the relationship between women’s empowerment and outcome variables adjusting for covariates variables regression with svy command to adjust for the DHS sampling design. Data were analyzed by STATA 14.” |
|  | In Table (1), no need to present "currently not using", the row for No in "Knowledge of fertile period", "heard about FP on radio", "TV", "newspaper" and label should be "women" in stead of "mother". | Thank you very much, we have revised it. |
|  | 6. PLOS authors have the option to publish the peer review history of their article ([what does this mean?](https://journals.plos.org/plosone/s/editorial-and-peer-review-process#loc-peer-review-history)). If published, this will include your full peer review and any attached files.   If you choose “no”, your identity will remain anonymous but your review may still be made public.   **Do you want your identity to be public for this peer review?** For information about this choice, including consent withdrawal, please see our [Privacy Policy](https://www.plos.org/privacy-policy).  Reviewer #1: No  Reviewer #2: No |  |
|  |  |  |
