# Supplementary material for: Women’s empowerment and contraceptive use: Recent evidence from ASEAN countries
Source: PLoS One. 2023 Jun 27;18(6):e0287442. doi: 10.1371/journal.pone.0287442 (PMC10298759; doi:10.1371/journal.pone.0287442)
Supplement: Attachment — Submitted filename: Response to Reviewers.docx [file pone.0287442.s003.docx]

To: Thae Maung Maung, MBBS, MSc (International Health), PhD

Academic Editor

PLOS ONE

Re: Revised Manuscript

Date:	23 January 2023

————————————————————————————————————

Dear Academic Editor

Thank you for your email dated 16 January 2023 with the reviewer comments for the manuscript titled “Women’s empowerment and contraceptive use: recent evidence from ASEAN countries” (Manuscript ID PONE-D-22-02451R1). As directed by your correspondence, we are submitting the revised manuscript with changes highlighted in red colour that addresses all reviewer comments and suggestions. Below, please find our responses to the reviewer’s comments. We thank you for the opportunity to submit this revised manuscript.

Comments to the Author

| **No.** | **Comment** | **Response** |
| --- | --- | --- |
|  | **Additional Editor Comments:** |  |
| 1. | Please include the following items when submitting your revised manuscript:   - A rebuttal letter that responds to each point raised by the academic editor and reviewer(s). You should upload this letter as a separate file labeled 'Response to Reviewers'. - A marked-up copy of your manuscript that highlights changes made to the original version. You should upload this as a separate file labeled 'Revised Manuscript with Track Changes'. - An unmarked version of your revised paper without tracked changes. You should upload this as a separate file labelled 'Manuscript'.   If you would like to make changes to your financial disclosure, please include your updated statement in your cover letter. Guidelines for resubmitting your figure files are available below the reviewer comments at the end of this letter. | Thank you very much for your suggestion. We have prepared the files as suggested. |
|  | **Reviewer's Responses to Questions** |  |
| 1. | If the authors have adequately addressed your comments raised in a previous round of review and you feel that this manuscript is now acceptable for publication, you may indicate that here to bypass the “Comments to the Author” section, enter your conflict of interest statement in the “Confidential to Editor” section, and submit your "Accept" recommendation.  Reviewer #1: All comments have been addressed | Thank you for the comments. |
| 2. | Is the manuscript technically sound, and do the data support the conclusions?  The manuscript must describe a technically sound piece of scientific research with data that supports the conclusions. Experiments must have been conducted rigorously, with appropriate controls, replication, and sample sizes. The conclusions must be drawn appropriately based on the data presented.  Reviewer #1: Partly | Thank you for the comments. |
| 3. | Has the statistical analysis been performed appropriately and rigorously?  Reviewer #1: No | Thank you for the comments. |
| 4. | Have the authors made all data underlying the findings in their manuscript fully available?  The [PLOS Data policy](http://www.plosone.org/static/policies.action#sharing) requires authors to make all data underlying the findings described in their manuscript fully available without restriction, with rare exception (please refer to the Data Availability Statement in the manuscript PDF file). The data should be provided as part of the manuscript or its supporting information, or deposited to a public repository. For example, in addition to summary statistics, the data points behind means, medians and variance measures should be available. If there are restrictions on publicly sharing data—e.g. participant privacy or use of data from a third party—those must be specified.  Reviewer #1: Yes | Thank you for the comments. |
| 5. | Is the manuscript presented in an intelligible fashion and written in standard English?  PLOS ONE does not copyedit accepted manuscripts, so the language in submitted articles must be clear, correct, and unambiguous. Any typographical or grammatical errors should be corrected at revision, so please note any specific errors here.  Reviewer #1: Yes | Thank you for the comments. |
| 6. | PLOS authors have the option to publish the peer review history of their article ([what does this mean?](https://journals.plos.org/plosone/s/editorial-and-peer-review-process#loc-peer-review-history)). If published, this will include your full peer review and any attached files.   If you choose “no”, your identity will remain anonymous but your review may still be made public.   **Do you want your identity to be public for this peer review?** For information about this choice, including consent withdrawal, please see our [Privacy Policy](https://www.plos.org/privacy-policy).  Reviewer #1: No | Thank you for the comments. |
|  | **Review Comments to the Author**  Reviewer #1: This revised manuscript assesses the relationship between measures of women's empowerment and contraceptive use in five ASEAN countries. The topic is important, and is understudied in the focal region. Below are comments that I hope will further improve the contribution of this manuscript: | Thank you very much for the suggestions. |
| 1. | Describing the countries included in the analysis. A Table with key national indicators and sample characteristics, for each included country would help to situate the analysis in regional context. | Thanks for the suggestion, we have added key national indicators for each country in table 1. |
| 2. | A clearer description of the outcome variable--contraceptive use is needed. What specific methods were asked in each country? Is the measure constructed to be comparable across settings? | Thank you, we have added the clearer description of the outcome variable—contraceptive use in line 147-153. |
| 3. | The conceptual framework should appear in the background, and measures for empowerment should align with Kabeer's framework, as presented in the background. For example, women's labor force participation in this model arguably is a resource for empowerment; whereas, disagreement with wive beating, decision-making, and "knowledge" are meaures of individual agency. These measures might be more clearly conceptually organized?  Also, whey is women's schooling not included as a resource for their empowerment in the model? | Thank you for the suggestion, we have added the conceptual framework which align with Kabeer’s framework as described in the background section.  Women's schooling is included in the component of women's knowledge as a measure of empowerment, this explained in the method section. |
| 4. | A much clearer description for how the empowerment variables are constructed is needed. For example, how are the 5 IPV attitudes items coded and combined. This is not described in any detail in the manuscript, and the construction of the other empowerment variables also need more detail. | Many thanks, we have added the clearer description for each empowerment variables in line 167-199. |
| 5. | A thorough editing of the manuscript is needed throughout for clarity of exposition. | Many thanks for the comment, we have edited throughout the text |
| 6. | The description of the sample sizes for each country should appear in the methods, with clarity about the original number of eligible women for each country and numbers of women dropped due to any missing data. | Thank you for the comment, we have added the description of the sample size with clarity of the original and actual sample size used in the study in line 124-134. |
| 7. | The description of the methods of analysis is very limited and needs more detail and clarity of writing. | Thank you for the comment we have added more detailed of analysis methods in line 217-221 |
| 8. | Interpretation of the findings needs some work. For example, these are cross-sectional surveys, and so it would be more appropriate to speak of higher/lower contraceptive use by covariates of interest, rather than increases/decreases. This interpretation also aligns with the associational nature of the findings from a cross-sectional study. | Thank you for the suggestion, we have revised in line 231-240 |
| 9. | More nuanced discussion of the similarities and differences in the findings across ASEAN settings would be helpful. | Many thanks for the improvement comment, we have added some explanation about the unique information of each countries to enrich the discussion. |

[NOTE: If reviewer comments were submitted as an attachment file, they will be attached to this email and accessible via the submission site. Please log into your account, locate the manuscript record, and check for the action link "View Attachments". If this link does not appear, there are no attachment files.]

While revising your submission, please upload your figure files to the Preflight Analysis and Conversion Engine (PACE) digital diagnostic tool, <https://pacev2.apexcovantage.com/>. PACE helps ensure that figures meet PLOS requirements. To use PACE, you must first register as a user. Registration is free. Then, login and navigate to the UPLOAD tab, where you will find detailed instructions on how to use the tool. If you encounter any issues or have any questions when using PACE, please email PLOS at [figures@plos.org](mailto:figures@plos.org). Please note that Supporting Information files do not need this step.
