# Supplementary material for: Women’s empowerment and contraceptive use: Recent evidence from ASEAN countries
Source: PLoS One. 2023 Jun 27;18(6):e0287442. doi: 10.1371/journal.pone.0287442 (PMC10298759; doi:10.1371/journal.pone.0287442)
Supplement: Attachment — Submitted filename: Response to Reviewers.docx [file pone.0287442.s004.docx]

To: Thae Maung Maung, MBBS, MSc (International Health), PhD

Academic Editor

PLOS ONE

Re: Revised Manuscript

Date:	24 May 2023

————————————————————————————————————

Dear Academic Editor

Thank you for your email dated 11 May 2023 with the reviewer comments for the manuscript titled “Women’s empowerment and contraceptive use: recent evidence from ASEAN countries” (Manuscript ID [PONE-D-22-02451R2). As directed by your correspondence, we are submitting the revised manuscript with changes highlighted in red colour that addresses all reviewer comments and suggestions. Below, please find our responses to the reviewer’s comments. We thank you for the opportunity to submit this revised manuscript.

Comments to the Author

| **No.** | **Comment** | **Response** |
| --- | --- | --- |
|  | **Additional Editor Comments:** |  |
| 1. | Please include the following items when submitting your revised manuscript:   - A rebuttal letter that responds to each point raised by the academic editor and reviewer(s). You should upload this letter as a separate file labeled 'Response to Reviewers'. - A marked-up copy of your manuscript that highlights changes made to the original version. You should upload this as a separate file labeled 'Revised Manuscript with Track Changes'. - An unmarked version of your revised paper without tracked changes. You should upload this as a separate file labelled 'Manuscript'.   If you would like to make changes to your financial disclosure, please include your updated statement in your cover letter. Guidelines for resubmitting your figure files are available below the reviewer comments at the end of this letter. | Thank you very much for your suggestion. We have prepared the files as suggested. |
| 2. | Journal Requirements:  Please review your reference list to ensure that it is complete and correct. If you have cited papers that have been retracted, please include the rationale for doing so in the manuscript text, or remove these references and replace them with relevant current references. Any changes to the reference list should be mentioned in the rebuttal letter that accompanies your revised manuscript. If you need to cite a retracted article, indicate the article’s retracted status in the References list and also include a citation and full reference for the retraction notice. | Thank you for your comments on our reference list in our paper. We appreciate your thorough analysis and would want to note that we have already checked that our reference list is entire, correct, and accurate. |
|  | **Reviewer's Responses to Questions** |  |
| 1. | If the authors have adequately addressed your comments raised in a previous round of review and you feel that this manuscript is now acceptable for publication, you may indicate that here to bypass the “Comments to the Author” section, enter your conflict of interest statement in the “Confidential to Editor” section, and submit your "Accept" recommendation.  Reviewer #3: All comments have been addressed | Thank you for the feedback |
| 2. | Is the manuscript technically sound, and do the data support the conclusions?  The manuscript must describe a technically sound piece of scientific research with data that supports the conclusions. Experiments must have been conducted rigorously, with appropriate controls, replication, and sample sizes. The conclusions must be drawn appropriately based on the data presented.  Reviewer #3: Yes | Thank you for the feedback |
| 3. | Has the statistical analysis been performed appropriately and rigorously?  Reviewer #3: Yes | Thank you for the feedback |
| 4. | Have the authors made all data underlying the findings in their manuscript fully available?  The PLOS Data policy requires authors to make all data underlying the findings described in their manuscript fully available without restriction, with rare exception (please refer to the Data Availability Statement in the manuscript PDF file). The data should be provided as part of the manuscript or its supporting information, or deposited to a public repository. For example, in addition to summary statistics, the data points behind means, medians and variance measures should be available. If there are restrictions on publicly sharing data—e.g. participant privacy or use of data from a third party—those must be specified.  Reviewer #3: Yes | Thank you for the feedback |
| 5. | Is the manuscript presented in an intelligible fashion and written in standard English?  PLOS ONE does not copyedit accepted manuscripts, so the language in submitted articles must be clear, correct, and unambiguous. Any typographical or grammatical errors should be corrected at revision, so please note any specific errors here.  Reviewer #3: No | Thank you for the valuable comment, we have made a correction through our manuscript, and we have done made the revision for improving our English written. |
| 6. | Review Comments to the Author  Please use the space provided to explain your answers to the questions above. You may also include additional comments for the author, including concerns about dual publication, research ethics, or publication ethics. (Please upload your review as an attachment if it exceeds 20,000 characters)  Reviewer #3: Authors adequately addressed previous comments.  However, the manuscript still needs thorough copy editing to remove few grammatical errors.  Please remove the subtitles in the Discussion section. | Thank you for the suggestion, we have removed all the subtitled in the discussion section. |
| 7. | PLOS authors have the option to publish the peer review history of their article (what does this mean?). If published, this will include your full peer review and any attached files.  If you choose “no”, your identity will remain anonymous but your review may still be made public.  Do you want your identity to be public for this peer review? For information about this choice, including consent withdrawal, please see our Privacy Policy.  Reviewer #3: No | Thank you very much for the feedback |

[NOTE: If reviewer comments were submitted as an attachment file, they will be attached to this email and accessible via the submission site. Please log into your account, locate the manuscript record, and check for the action link "View Attachments". If this link does not appear, there are no attachment files.]

While revising your submission, please upload your figure files to the Preflight Analysis and Conversion Engine (PACE) digital diagnostic tool, <https://pacev2.apexcovantage.com/>. PACE helps ensure that figures meet PLOS requirements. To use PACE, you must first register as a user. Registration is free. Then, login and navigate to the UPLOAD tab, where you will find detailed instructions on how to use the tool. If you encounter any issues or have any questions when using PACE, please email PLOS at [figures@plos.org](mailto:figures@plos.org). Please note that Supporting Information files do not need this step.
